# Relationship Between Frailty and Risk of Falls Among Hospitalised Older People with Cardiac Conditions: An Observational Cohort Study

**DOI:** 10.3390/nursrep15030100

**Published:** 2025-03-15

**Authors:** Noel Rivas-González, María López, Belén Martín-Gil, Mercedes Fernández-Castro, María José Castro, J. Alberto San Román

**Affiliations:** 1 Continuing Education Department, Valladolid University Clinical Hospital, 47005 Valladolid, Spain; nrivas@saludcastillayleon.es; 2 Faculty of Nursing, University of Valladolid, 47003 Valladolid, Spain; mariajose.castro@uva.es; 3 GIR Research Group on Multidisciplinary Assessment and Intervention in Health Care and Sustainable Lifestyles, University of Valladolid, 47003 Valladolid, Spain; 4 Department of Nursing Care Information Systems, Valladolid University Clinical Hospital, 47005 Valladolid, Spain; bmartingi@saludcastillayleon.es; 5 Research Support Unit, Valladolid University Clinical Hospital, 47005 Valladolid, Spain; mefernandezc@saludcastillayleon.es; 6 Cardiology Department, Valladolid University Clinical Hospital, 47005 Valladolid, Spain; asanroman@secardiologia.es; 7 Centro de Investigación en Red de Enfermedades Cardiovasculares (CIBERCV), 28029 Madrid, Spain

**Keywords:** frailty, accidental falls with risk fall, older adults, cardiovascular diseases, hospitalisation

## Abstract

**Background/Objective**: Ageing favours the onset of cardiovascular diseases, frailty, and risk of falls. In the hospital setting, 47.7% of patients may be frail, and the incidence of falls may be as high as five per thousand. This study seeks to determine the relationship between frailty, risk of falls, and length of hospital stays in hospitalised older adults with heart disease. **Methods**: An observational study was conducted of a cohort of patients aged ≥60 years admitted to a cardiology unit (2022–2024). Frailty was assessed using Fried’s phenotype, risk of falls using the J.H. Downton scale, and level of dependency using the Barthel index. Clinical variables, anthropometric measurements, and length of stay were analysed. Statistical analysis: quantitative variables were expressed as means and standard deviations, and categorical variables as frequencies. Associations were analysed using Student’s *t*-tests, chi-squared tests, and Kruskal–Wallis tests for comparisons of three or more groups. Relationships between frailty, risk of falls, and other variables were examined using univariate binary logistic regression, with a 95% confidence interval and statistical significance set at *p* < 0.05. **Results**: A total of 144 patients were recruited (mean age = 73.08 years [SD = 7.95]) (women = 33.30%). Frailty was associated with waist circumference in men (*p* = 0.01) and diastolic blood pressure in women (*p* = 0.05). Frailty was further linked to Downton scores (odds ratio [OR] = 1.565; 95% CI: 1.156–2.120; *p* = 0.004), age (OR = 1.114; 95% CI: 1.058–1.173; *p* = 0.000), Barthel index (OR = 0.902; 95% CI: 0.854–0.953; *p* = 0.000), and length of stay (OR = 1.101; 95% CI: 1.021–1.186; *p* = 0.012). **Conclusions**: Frailty appears to be related to Downton scores and impacts the length of hospital stays in older adults hospitalised with cardiac conditions.

## 1. Introduction

Frailty syndrome, cardiovascular diseases (CVDs), and falls are three of the most prevalent health issues among older adults [1], with significantly different outcomes between sexes [2,3,4].

The ageing process begins at 60 years of age, a stage marked by declining physical condition and an increased risk of disease. Globally, the number of people aged 60 and above reached 1 billion in 2019 and is projected to rise to 2.5 billion by 2050 [5].

CVDs are one of the most common conditions worldwide during this period, directly linked to morbidity, disability, and mortality [6]. The risk of CVD is particularly elevated among women due to the physiological changes associated with menopause [7,8].

In later life, an imbalance in homeostasis can lead to the emergence of frailty syndrome, first defined by Linda Fried et al. as a physical phenotype in 2001. Various tools for identifying frailty exist in the literature, with Fried’s five-criteria scale being among the most widely used [9].

Frailty has been associated with adverse outcomes such as increased risk of hospitalisation, falls, and mortality, all of which lead to higher healthcare costs [10].

Ongoing research into defining frailty and developing tools to address its multifactorial nature reveals varying prevalence rates depending on the study setting. Doody et al. (2023) reported a frailty prevalence of 10.7% among community-dwelling older adults, compared to 47.4% among hospitalised older patients [1]. Despite having a longer life expectancy, women are more frequently affected by frailty [2], with a prevalence of 9.6% compared to 5.4% in men [11].

Xu, Ou, and Li provided evidence that CVD, frailty, and hypertension increase the risk of falls [12]. Similarly, a study in the United Kingdom found that pre-frail and frail patients had a 47% and 15% higher risk of developing CVD, respectively, compared to non-frail patients [13]. Atrial fibrillation is the most common type of cardiac arrhythmia in older adults, with the incidence estimated to increase progressively from the age of 50 in men and 60 in women [14]. Atrial fibrillation in hospitalised patients has been strongly associated with frailty (OR = 4.09, 95%CI: 1.51–11.07) [15].

Other researchers have linked CVD to an elevated risk of mortality (relative risk [RR] = 1.81; 95% CI: 1.67–1.97) among fall-related incidents, with age being a contributing factor [16,17].

The multifactorial concept of frailty has led to the identification of associated factors such as advanced age, polypharmacy, or female sex, among others. In the same way, comorbidities have been identified that can increase the risk of presenting with frailty. Similarly, comorbidities that may increase the risk of frailty have been identified, such as diabetes or previous falls [18].

Falls compromise personal safety and are more common among older adults [19], with a higher prevalence among women [20]. In hospital settings, falls are the most frequent adverse event, with an incidence of 3–5 falls per 1000 patient-days and an estimated annual occurrence of 700,000 to 1 million cases [21,22].

Preventing falls has been a key area of study. Multifactorial programmes such as 6-PACK and Best Practice Spotlight Organisations (BPSO^®^) include guidelines like the Prevention of Falls and Fall Injuries Best Practice Guideline [23,24]. A common recommendation across these programmes is to identify patients at risk of falling so as to implement targeted interventions [25,26].

The region of Castile & León in Spain is a geographical area with demographic indicators that reflect an ageing, a longevity, and a dependency higher than the rest of the Spanish population, which conditions its service strategy in relation to dependency, multiple pathology, and chronicity.

The interplay between CVD, frailty, and falls poses a significant challenge to therapeutic actions for these patients. These challenges are further influenced by sex differences and the traditional threshold for ageing being set at 65 years, even though physiological changes begin at 60 years. Addressing these factors could have a profound impact on population health and the economic resources of healthcare systems. For these reasons, this study aimed to analyse the relationship between frailty, risk of falls, and length of hospital stay in hospitalised older adults with cardiac conditions.

## 2. Materials and Methods

### 2.1. Study Design, Setting, and Sampling

An observational pilot cohort study was conducted in a tertiary hospital within the public network of the Spanish National Health System. Recruitment took place between March 2022 and September 2024, involving patients admitted to a conventional cardiology hospitalisation unit. Individuals aged ≥60 years who were conscious and oriented were included. Patients with cognitive impairment, admission of less than 48 h, bed rest, or where not all the data required for the study could not be collected were excluded from the study.

The study is reported according to STROBE reporting guidelines for observational research.

The study participants were selected using a non-probabilistic convenience sampling method in the cardiology unit. Patients were recruited as they were consecutively admitted to the unit.

### 2.2. Instruments

#### 2.2.1. Fall Risk Assessment

Fall risk screening was conducted using the BPSO^®^ programme, introduced in Spain in 2010 through the Carlos III Health Institute under the Ministry of Science, Innovation, and Universities. The programme was implemented in the study hospital in 2018, with the Prevention of Falls and Fall Injuries guideline being adopted in 2019. Fall risk was evaluated using the J.H. Downton scale, which assesses five dimensions: previous falls, medication, sensory deficits, mental state, and gait [27]. The J.H. Downton scale has a sensitivity of 0.58 and specificity of 0.63. Scores were interpreted as follows: 0 points = no risk, 1–2 points = low/moderate risk, and ≥3 points = high risk [28]. The J.H. Downton falls risk scale was selected as a tool for the detection of the risk of falls by the Regional Health Management of Castile & León in consensus with the 14 hospitals of the region.

#### 2.2.2. Frailty Identification

Patients classified as frail were identified using Fried’s five criteria [9]:

1. Unintentional weight loss >4.5 kg or >5.0% (in the past year).
2. Generalised exhaustion (low energy and endurance measured by the CES-D depression scale [29]).
3. Weakness (handgrip strength adjusted for sex and body mass index using a hand dynamometer, the JAMAR**^®^** Plus (Performance Health Supply, Inc., Cedarburg, WI, USA) digital hand-held dynamometer with LCD display (0–90 kg)).
4. Walking speed (time to cover 4.57 m adjusted for sex and height).
5. Weekly physical activity (Minnesota Leisure Time Activity Questionnaire [MLTAQ] stratified by sex; men: 383 kcal/week, women: 270 kcal/week [30]).

Patients were classed based on the number of criteria they met:

- Frail: three or more criteria.
- Pre-frail: one or two criteria.
- Non-frail: no criteria.

#### 2.2.3. Functional Dependency

Functional dependency was assessed using the Barthel index, which has a Cronbach’s alpha of 0.70 [31]. Scores were categorised in the following manner:

- 0–20: total dependence;
- 21–60: severe dependence;
- 61–90: moderate dependence;
- 91–99: minimal dependence;
- 100: independence.

### 2.3. Description of Variables

The following variables were considered:

- Sociodemographic variables: sex (biological attribute); age.
- Dependent variable: fall risk (yes/no); score on the J.H. Downton scale.
- Independent variables; the following independent variables were considered:

1. Frailty status.
2. Clinical variables: main diagnosis (coronary heart disease; infectious endocarditis; heart failure; arrhythmias; heart transplant; and valvular diseases); diabetes mellitus (yes/no); readmission to the cardiology unit (yes/no); presence of dyspnoea at admission (yes/no); presence of chest pain at admission (yes/no); systolic blood pressure (SBP; mmHg); diastolic blood pressure (DBP; mmHg); heart rate (HR; beats per minute); oxygen saturation (SpO_2_; %); and blood glucose (mg/dL).
3. Anthropometric variables: height (cm); weight (kg); waist circumference (cm); body mass index (BMI; kg/m^2^: overweight (≥25 kg/m^2^), obesity (≥30 kg/m^2^)).
4. Length of stay: number of hospitalisation days.

### 2.4. Procedure

A maximum of three days from the day of admission was established for data collection. Patients meeting the inclusion criteria were informed about the study, and after obtaining their informed consent, the following data were extracted from their electronic health records: sex, age, primary diagnosis, diabetes mellitus status, readmission to the cardiology unit, presence of dyspnoea, SBP, DBP, HR, SpO_2_, and blood glucose levels. At admission, the degree of dependency was systematically assessed using the Barthel index, and fall risk was screened using the J.H. Downton scale, with scores of 1 or higher considered positive.

Anthropometric measures such as height, weight, and BMI were recorded, and Fried’s scale was applied to identify frail patients. The length of stay in days was documented for all participants upon hospital discharge.

### 2.5. Ethical Considerations

The anonymity and confidentiality of participant data were safeguarded using Research Electronic Data Capture (REDCap) software (https://project-redcap.org/), in compliance with the European Regulation 2016/679 of the European Parliament and of the Council of 27 April 2016 and Spanish Organic Law 3/2018, of 5 December, on the Protection of Personal Data and the Guarantee of Digital Rights. The study was conducted in accordance with the Declaration of Helsinki, and the protocol was approved by the relevant Valladolid Ethics Committee for Research with medicinal products (ECRmp) involving humans under the reference code PI-20-1612 on 23 January 2020.

### 2.6. Statistical Analysis

Data normality was assessed using the Kolmogorov–Smirnov test, kurtosis, and skewness. Quantitative variables were presented as means and standard deviations (SDs), while categorical variables were expressed as frequency distributions. Quantitative variables were compared using Student’s *t*-test, categorical variables with Pearson’s chi-squared test, and comparisons among three groups using the Kruskal–Wallis test. Homogeneity of variance was evaluated using Levene’s test.

Univariate binary logistic regression was employed to identify factors associated with frailty and fall risk, with adjusted odds ratios (ORs) calculated for the included variables. A confidence interval of 95% and a statistical significance level of *p* < 0.05 were applied.

Statistical analyses were performed using IBM^®^ SPSS^®^ Statistics v.26 software (SPSS, Inc., Chicago, IL, USA).

## 3. Results

### 3.1. Descriptive Analysis

During the study period, 148 patients were recruited, of which 4 patients were excluded from the study as the necessary data collection could not be completed (33.30% women, *n* = 48; 66.70% men, *n* = 96), with a mean age of 73.08 years (SD = 7.95). The most prevalent diagnosis was coronary heart disease (61.11%). Diabetes mellitus was present in 36.11% of patients, while 35.42% reported chest pain at admission, and 30.56% presented with dyspnoea.

Regarding frailty, 33.30% of patients were classed as frail. Fall risk was identified in 97.22% of participants, with 36.81% of the total sample considered at high or very high risk (Table 1).

The mean Downton score indicated a low/moderate fall risk (2.24 points; SD = 1.25). The Barthel index showed a mean score of 95.76 points (SD = 9.28), reflecting a minimal level of dependency in most patients. The mean BMI fell within the overweight category ($\bar{X}$ = 27.69 kg/m^2^; SD = 4.35), and the mean waist circumference exceeded 100 cm. The mean overall hospital stay was 8.66 days (SD = 5.56), while patients at high fall risk experienced a longer mean stay of 9.71 days (SD = 7.39) (Table 2).

After analysing the data by sex, the mean age was similar between men and women, as was the Downton score. Women were proportionally frailer and had a lower mean score on the Barthel index (93.54 points; SD = 12.80). There were no differences in mean BMI (men: $\bar{X}$ = 27.71 kg/m^2^; SD = 4.09; women: $\bar{X}$ = 27.67 kg/m^2^; SD = 4.89). However, the mean waist circumference was larger in men than in women. The mean length of the hospital stay was longer among men ($\bar{X}$ = 9.40 days; SD = 6.13) compared to women ($\bar{X}$ = 7.11 days; SD = 3.78). The mean hospital stay duration in those patients with frailty was higher, both in the overall sample ($\bar{X}$ = 10.65; SD = 6.98) and in the sex analysis (men: $\bar{X}$ = 12.35; SD = 8.24 vs. women: $\bar{X}$ = 7.86; SD = 2.86) (Table 2).

The most prevalent diagnosis was coronary heart disease. The proportion of patients with diabetes was higher among men (39.58%). Fall risk was greater among frail and pre-frail women compared to other groups (37.50% and 43.80%, respectively) (Table 3).

When analysing the frailty distribution within the high/very high fall risk group, 45.30% of the patients were classified as frail. Women in this group exhibited a higher percentage of pre-frailty compared to men (42.10% vs. 17.60%). This group of patients had a mean hospital stay of 12.06 days (SD = 9.05).

According to the J.H. Downton scale, the most frequently used category of medications among patients was other medications (27.10%), followed by non-diuretic antihypertensives taken in combination with other drugs (16.00%). Notably, 99.30% of the patients were taking at least one medication during the assessment (Table 4).

### 3.2. Comparison Between Frailty Status and Demographic and Clinical Variables

A comparative analysis between categorical variables and frailty status revealed statistically significant differences in gait (assessed within the Downton scale) (χ2 = 25.14; df = 10; *p* < 0.00); social risk (χ2 = 8.88; df = 2; *p* < 0.01); and gender (χ2 = 5.99; df = 2; *p* < 0.05). However, no significant differences were found with risk of falls (χ2 = 0.62; df = 2; *p* = 0.73).

Regarding quantitative variables, significant differences were observed between non-frail and frail patients in diastolic blood pressure (t = 2.19; df = 92; *p* = 0.03); waist circumference (t = −2.22; df = 91; *p* = 0.02); length of hospital stay (t = −2.74; df = 92; *p* = 0.00); and height (t = 2.96; df = 92; *p* < 0.00).

Among women, a significant association was observed between frailty status (frail and non-frail) and diastolic blood pressure (t = −2.06; df = 25; *p* = 0.05). In men, waist circumference was significantly associated with frailty status (frail vs. non-frail) (t = −2.61; df = 65; *p* = 0.01).

In the analysis of the three frailty groups (frail, pre-frail, and non-frail), statistically significant differences were found in the following variables: Downton score (H = 7.73; df = 2; *p* = 0.02); Barthel index (H = 25.68; df = 2; *p* = 0.00); length of hospital stay (H = 6.97; df = 2; *p* = 0.03); waist circumference (H = 6.78; df = 2; *p* = 0.03); age (H = 19.75; df = 2; *p* = 0.00); and height (H = 6.75; df = 2; *p* = 0.03) (Table 5).

Among women, a significant association was observed between frailty status and the Barthel index in the Pre-frail–Frail group (H = 7.71; *p* = 0.04). For men, a significant association was found in the Frail–Non-frail group (H = 14.00; *p* < 0.00).

### 3.3. Logistic Regression Analysis

No significant association was found between risk of falls and frailty status (OR: 0.959; 95% CI: 0.058–15.752; *p* = 0.977). However, waist circumference was significantly associated with risk of falls (OR: 1.063; 95% CI: 1.003–1.125; *p* = 0.038).

With respect to age, for every year, older patients are 1.114 times more likely to be frail.

Frailty status was directly related to Downton scores, age, Barthel index, oxygen saturation (SpO_2_), diastolic blood pressure, and waist circumference. No statistically significant relationship was observed with sex (Table 6).

When examining the relationship between frailty and specific items from the Downton scale, the use of diuretics, sensory deficits in the extremities, and gait were significantly associated with frailty (Table 7).

A statistically significant relationship was observed between coronary heart disease and frailty status. Coronary artery disease was the only clinical diagnosis at admission that was inversely related to frailty status in a statistically significant manner. Patients admitted with coronary artery disease were 5.64 times less likely to be frail (Table 8).

## 4. Discussion

Scientific cardiology societies should prioritise including frail patients with cardiovascular disease (CVD) in their research projects in order to optimise their clinical management, particularly considering the existing link between frailty and risk of falls [32,33].

Our study identified a relationship between frailty status and scores on the J.H. Downton scale, with frail hospitalised cardiac patients scoring higher and, thus, facing a greater risk of falls. However, no significant association was found between frailty status and binary fall risk (Yes/No), as determined by the same scale. This finding aligns with a study conducted on a rural Ecuadorian population aged ≥60 years (OR: 0.63; 95% CI: 0.29–1.36; *p* = 0.237), but contrasts with Yang et al.’s meta-analysis (RR: 1.48; 95% CI: 1.27–1.73; *p* < 0.05) [14,34]. These differences could be attributed to the use of varied tools for assessing both frailty and fall risk.

The limited predictive value of the J.H. Downton scale has been noted by other researchers, who have emphasised the critical role of nurses’ clinical judgement. In hospital settings, the Downton scale may overidentify fall risk, as the threshold begins at just 1 point. Despite its widespread use, pairing it with another tool that captures the multifactorial nature of fall risk is recommended [28,35,36].

Although no statistically significant relationship was found between frailty and fall risk by sex, differences were observed in related factors such as blood pressure and waist circumference. Previous studies have linked frailty in women to higher mortality (hazard ratio [HR]: 0.65; 95% CI: 0.58–0.74; *p* < 0.001) [37].

Larger waist circumference in this study was associated with increased fall risk, consistent with Bu et al.’s findings (OR: 0.98; 95% CI: 0.96–1.00; *p* = 0.017) [38]. Polypharmacy is also a well-documented fall risk factor in CVD patients. In this study, frail patients were more likely to use diuretics than their non-frail counterparts. Diuretics are among the drugs most commonly linked to fall risk in this population, as highlighted by the American Heart Association [32].

The prevalence of frailty in our study was lower than reported in other non-community settings but higher than in community settings [1,39]. Differences in frailty assessment tools, population groups, and identification criteria continue to hinder the development of standardised protocols [40].

In one out of three patients with diabetes, frailty status could be identified, although no relationship between these two conditions could be established, as in the study by Xu et al. [12]. However, other authors have identified diabetes as a factor associated with frailty that may increase the risk of developing it (RR: 1.10, 95% CI: 1.01–1.20, *p* = 0.04). Among the CVD diagnoses included in the study, frailty was only significantly associated with coronary heart disease. This contrasts with findings from Wang, Hu, and Wu, who found no overall relationship between frailty and CVD (RR: 1.00; 95% CI: 0.92–1.09; *p* = 0.95) [18]. Liperoti et al. estimated that nearly one-fifth of patients with ischaemic heart disease were frail but did not establish a clear link [41]. While frailty is often associated with heart failure [42], this was not statistically significant in our study. Diastolic blood pressure was inversely related to frailty, suggesting that frail patients tended to have lower diastolic pressures. This finding aligns with previous studies [43], although Vetrano et al. reported a relationship between frailty and hypertension (pooled OR: 1.33; 95% CI: 0.94–1.89; I2 = 79.2%) [44]. Age also emerged as a significant factor, with increasing age linked to higher frailty risk, consistent with prior studies ([OR: 1.15; 95% CI: 1.09–1.21; *p* < 0.001]; [OR: 3.910; 95% CI: 2.021–5.402; *p* < 0.05]) [34,45].

Frail patients also experienced longer hospital stays, with men staying the longest. When combined with fall risk, hospital stays extended to a mean of 12.06 days, likely increasing institutional costs [10].

Through the BPSO programme of the RNAO, the J.H. Downton scale has been implemented since 2019 in the 14 hospitals of the Castile & León region to assess the risk of falls in hospitalised patients. It is considered that patients with a score of 1 are already at risk, and it would be desirable to modify the criteria for risk designation or opt for another scale that fits the hospital setting. This will contribute to the design of new care management strategies in terms of fall risk detection and the identification of frailty in the hospital setting, increasing patient safety and deepening this line of research for the development of specific care plans to facilitate a more sustainable health system.

### 4.1. Limitations

The main limitation of this study was the sample size; the participants were selected through convenience sampling. A larger sample would have allowed for greater generalisability of results. However, the study objectives were successfully addressed. Future studies should examine factors influencing the relationship between frailty and fall risk for each clinical diagnosis and include a wider range of hospital settings.

#### Future Lines of Research

Larger, more diverse samples are essential to generalise findings and uncover the complex relationship between frailty, risk of falls, and cardiovascular conditions. Developing multifactorial models that combine clinical and demographic variables could transform personalised care for these patients.

## 5. Conclusions

Older frail adults hospitalised with cardiac disease have longer average hospital stays. Frailty was directly associated with Downton scores in hospitalised older adult patients with cardiac conditions. In this study, 33.30% of patients were classed as frail, and 97.22% were at risk of falls. Women at high fall risk were proportionally frailer than men in the same situation. Frail patients also had longer hospital stays than their non-frail counterparts.

As the older population grows worldwide, the number of individuals with CVD who are frail and at risk of falls is expected to increase. Identifying shared characteristics within this population can help standardise assessment tools and improve clinical management, leading to better outcomes for patients and institutions alike. Combining frailty and fall risk scales in hospitalised older adults with cardiac conditions can refine prognostic accuracy, address comorbidities, and reduce hospital stays. Tailoring interventions to the differences between men and women is equally important for effective management.

## Figures and Tables

**Table 1:** Sample description.

*n* = 144			Men *n* = 96 Women *n* = 48	Frail *n* = 48	Non-Frail *n* = 46	Pre-Frail *n* = 50		
				*n*	*n*	*n*	Total	% Total
Diagnosis	Arrhythmias	Sex	Male	3	3	1	7	4.86%
			Female	2	1	5	8	5.56%
			Total	5	4	6	15	10.42%
	Infectious endocarditis	Sex	Male	1	0	1	2	1.39%
			Female	0	0	0	0	0.00%
			Total	1	0	1	2	1.39%
	Heart failure	Sex	Male	7	8	6	21	14.58%
			Female	5	1	2	8	5.56%
			Total	12	9	8	29	20.14%
	Coronary heart disease	Sex	Male	15	26	20	61	42.36%
			Female	8	7	12	27	18.75%
			Total	23	33	32	88	61.11%
	Heart transplant	Sex	Male	1	0	0	1	0.69%
			Female	0	0	0	0	0.00%
			Total	1	0	0	1	0.69%
	Valvular disease	Sex	Male	3	0	1	4	2.78%
			Female	3	0	2	5	3.47%
			Total	6	0	3	9	6.25%
Readmission	NO	Sex	Male	23	34	26	83	57.64%
			Female	15	8	19	42	29.17%
			Total	38	42	45	125	86.81%
	YES	Sex	Male	7	3	3	13	9.03%
			Female	3	1	2	6	4.17%
			Total	10	4	5	19	13.19%
Diabetes mellitus	NO	Sex	Male	16	27	15	58	40.28%
			Female	12	7	15	34	23.61%
			Total	28	34	30	92	63.89%
	YES	Sex	Male	14	10	14	38	26.39%
			Female	6	2	6	14	9.72%
			Total	20	12	20	52	36.11%
Chest pain	NO	Sex	Male	19	23	22	64	44.44%
			Female	12	5	12	29	20.14%
			Total	31	28	34	93	64.58%
	YES	Sex	Male	11	14	7	32	22.22%
			Female	6	4	9	19	13.19%
			Total	17	18	16	51	35.42%
Dyspnoea	NO	Sex	Male	18	28	21	67	46.53%
			Female	12	7	14	33	22.92%
			Total	30	35	35	100	69.44%
	YES	Sex	Male	12	9	8	29	20.14%
			Female	6	2	7	15	10.42%
			Total	18	11	15	44	30.56%
Risk of falls (Downton)	NO	Sex	Male	0	2	1	3	2.08%
			Female	1	0	0	1	0.69%
			Total	1	2	1	4	2.78%
	YES	Sex	Male	30	35	28	93	64.58%
			Female	17	9	21	47	32.64%
			Total	47	44	49	140	97.22%
High/very high risk of falls (Downton > 3)	YES	Sex	Male	16	12	6	34	35.42%
			Female	8	3	8	21	39.58%
			Total	24	15	14	53	36.81%
Low/moderate risk of falls (Downton 1–2)	YES	Sex	Male	14	23	22	59	61.46%
			Female	9	6	13	16	33.33%
			Total	23	29	35	87	60.42%

**Table 2:** Descriptive statistics of quantitative variables in the total sample, by frailty status, according to Fried’s criteria and sex.

	*n* = 144	Frail *n* = 48	Non-Frail *n* = 46	Pre-Frail *n* = 50	Sex Male(M) = 96 Female (F) = 48		Frail M(31.30%) F(37.50%)	Non-Frail M(38.50%) F(18.80%)	Pre-Frail M(30.20%) F(43.80%)
Mean ($\bar{X}$) Standard deviation (SD)									
Age (years)	73.08 7.95	77.08 8.09	72.35 6.52	69.92 7.50	M	73.14 7.94	76.63 8.24	73.65 6.49	68.86 7.54
					F	72.98 8.06	77.83 8.01	67 3.04	71.38 7.38
Downton (points)	2.24 1.25	2.69 1.49	1.98 1.16	2.04 0.95	M	2.25 1.26	2.93 1.55	1.92 1.04	1.97 0.91
					F	2.21 1.24	2.28 1.32	2.22 1.64	2.14 1.01
Barthel index (points)	95.76 9.28	90.83 12.17	98.91 4.20	97.60 7.51	M	96.88 6.70	92.83 9.35	98.65 4.66	98.79 2.88
					F	93.54 12.80	87.5 15.55	100 0	95.95 11.02
Oxygen saturation (%)	94.99 2.58	94.38 2.92	95.52 1.75	95.10 2.80	M	94.85 2.83	94.1 3.5	95.41 1.80	94.93 3.05
					F	95.27 2.01	94.83 1.54	96 1.50	95.33 2.48
Heart rate (bpm)	73.47 16.34	73.56 15.48	75.17 18.36	71.80 15.29	M	71.97 16.31	73.1 17	72.19 16.79	70.52 15.39
					F	76.46 16.14	74.33 12.98	87.44 20.40	73.57 15.35
Systolic blood pressure (mmHg)	124.17 19.59	125.60 21.08	123.20 19.82	123.70 18.18	M	125.84 18.34	126.7 17.65	126.81 19.67	123.72 17.73
					F	120.83 21.71	123.78 26.29	108.33 12.61	123.67 19.23
Diastolic blood pressure (mmHg)	70.84 10.21	68.33 11.21	72.65 7.42	71.58 11.10	M	71.92 9.96	69.87 11.24	72.73 7.36	73 11.37
					F	68.69 10.48	65.78 11.01	72.33 8.12	69.62 10.68
Blood glucose (mg/dl)	134.62 52.78	137.08 61.79	135.46 54.86	131.48 41.07	M	134.91 49.35	134.87 54.96	134.46 50.57	135.52 42.95
					F	134.04 59.61	140.78 73.35	139.56 73.47	125.9 38.64
Waist circumference (cm)	102.96 12.83	106.23 12.14	101.25 9.25	101.45 15.65	M	104.83 10.82	108 11.32	101.58 8.78	105.71 11.82
					F	99.13 15.62	103.12 13.24	99.89 11.46	95.57 18.48
Length of stay (days)	8.66 5.56	10.65 6.98	7.03 4.00	8.30 4.79	M	9.40 6.13	12.35 8.24	7.33 4.29	9.14 5.64
					F	7.11 3.78	7.86 2.53	5.71 2.13	7.42 4.47
Height (cm)	164.44 7.87	162.43 7.81	166.71 6.02	164.28 8.94	M	168.15 5.81	166.48 5.58	168.34 4.93	169.62 6.76
					F	157.02 5.99	155.67 6.17	160 5.66	156.9 5.79
Weight (kg)	74.99 13.56	74.91 12.96	75.25 10.32	74.82 16.67	M	78.36 12.76	78.7 11.81	76.28 9.76	80.66 16.57
					F	68.25 12.68	68.6 12.62	70.98 12.01	66.77 13.39
BMI (kg/m^2^)	27.69 4.35	28.46 4.82	27.09 3.54	27.51 4.54	M	27.71 4.09	28.51 4.62	26.94 3.37	68.86 7.54
					F	27.67 4.89	28.38 5.27	27.72 4.34	71.38 7.38

**Table 3:** Distribution of total categorical variables by sex and frailty status.

Categorical Variables		% *n* = 144	Sex (Men: *n* = 96; Women: *n* = 48)	% Within Sex	Frail	Non-Frail	Pre-Frail
Diagnosis	Arrythmias	10.42%	Male	7.29%	3.13%	3.13%	1.04%
			Female	16.67%	4.17%	2.08%	10.42%
			Total		7.29%	5.21%	11.46%
	Infectious endocarditis	1.39%	Male	2.08%	1.04%	0.00%	1.04%
			Female	0.00%	0.00%	0.00%	0.00%
			Total		1.04%	0.00%	1.04%
	Heart failure	20.14%	Male	21.88%	7.29%	8.33%	6.25%
			Female	16.67%	10.42%	2.08%	4.17%
			Total		17.71%	10.42%	10.42%
	Coronary heart disease	61.11%	Male	63.54%	15.63%	27.08%	20.83%
			Female	56.25%	16.67%	14.58%	25.00%
			Total		32.29%	41.67%	45.83%
	Heart transplant	0.69%	Male	1.04%	1.04%	0.00%	0.00%
			Female	0.00%	0.00%	0.00%	0.00%
			Total		1.04%	0.00%	0.00%
	Valvular disease	6.25%	Male	4.17%	3.13%	0.00%	1.04%
			Female	10.42%	6.25%	0.00%	4.17%
			Total		9.38%	0.00%	5.21%
Readmission	NO	86.81%	Male	89.58%	23.96%	35.42%	27.08%
			Female	87.50%	31.25%	16.67%	39.58%
			Total		55.21%	52.08%	66.67%
	YES	13.19%	Male	13.54%	7.29%	3.13%	3.13%
			Female	12.50%	6.25%	2.08%	4.17%
			Total		13.54%	5.21%	7.29%
Diabetes mellitus	NO	63.89%	Male	60.42%	16.67%	28.13%	15.63%
			Female	70.83%	25.00%	14.58%	31.25%
			Total		41.67%	42.71%	46.88%
	YES	36.11%	Male	39.58%	14.58%	10.42%	14.58%
			Female	29.17%	12.50%	4.17%	12.50%
			Total		27.08%	14.58%	27.08%
Chest pain	NO	64.58%	Male	66.67%	19.79%	23.96%	22.92%
			Female	60.42%	25.00%	10.42%	25.00%
			Total		44.79%	34.38%	47.92%
	YES	35.42%	Male	33.33%	11.46%	14.58%	7.29%
			Female	39.58%	12.50%	8.33%	18.75%
			Total		23.96%	22.92%	26.04%
Dyspnoea	NO	69.44%	Male	69.79%	18.75%	29.17%	21.88%
			Female	68.75%	25.00%	14.58%	29.17%
			Total		43.75%	43.75%	51.04%
	YES	30.56%	Male	30.21%	12.50%	9.38%	8.33%
			Female	31.25%	12.50%	4.17%	14.58%
			Total		25.00%	13.54%	22.92%
Risk of falls (Downton)	NO	2.78%	Male	3.13%	0.00%	2.08%	1.04%
			Female	2.08%	2.08%	0.00%	0.00%
			Total		2.08%	2.08%	1.04%
	YES	97.22%	Male	96.98%	31.25%	36.46%	29.17%
			Female	97.92%	35.42%	18.75%	43.75%
			Total		66.67%	55.21%	72.92%

**Table 4:** Distribution of the most common medications used by patients assessed with the J.H. Downton scale.

Type of Medication	% (*n* = 144)
Antihypertensives	2.80
Non-diuretic antihypertensives	14.60
Antidepressants	0.70
Antiparkinsonians	2.10
Diuretics	3.50
Tranquillisers/sedatives	6.30
Diuretics and antihypertensives	3.50
Diuretics and non-diuretic antihypertensives	3.50
Diuretics and antidepressants	0.70
Diuretics and other medications	2.80
Other medications and non-diuretic antihypertensives	16.00
Tranquillisers/sedatives and non-diuretic antihypertensives	1.40
Tranquillisers/sedatives and other medications	1.40
Tranquillisers/sedatives, other medications, and non-diuretic antihypertensives	2.10
Diuretics, other medications, and non-diuretic antihypertensives	7.30
Tranquillisers/sedatives, antiparkinsonians, antidepressants, other medications, and non-diuretic antihypertensives	0.70
Other medications	27.10
None	3.50

**Table 5:** Association between frailty groups and demographic and clinical variables.

Fried	Variable	Test Statistic	Dev. Error	Dev. Test Statistic	*p*-Value
Non-frail—Pre-frail	Downton score	−3.70	8.22	−0.45	0.65
Non-frail—Frail		21.45	8.30	2.58	0.01
Pre-frail—Frail		17.74	8.13	2.18	0.03
Frail—Pre-frail	Barthel index	−24.58	6.64	−3.69	0.00
Frail—Non-frail		−32.92	6.78	−4.85	0.00
Pre-frail—Non-frail		8.34	6.72	1.24	0.21
Non-frail—Pre-frail	Length of hospital stay	−8.67	7.43	−1.17	0.24
Non-frail—Frail		19.58	7.43	2.64	0.00
Pre-frail—Frail		10.90	7.43	1.47	0.14
Pre-frail—Non-frail	Waist circumference	1.00	8.46	0.12	0.91
Pre-frail—Frail		19.65	8.41	2.34	0.02
Non-frail—Frail		18.65	8.59	2.17	0.03
Pre-frail—Non-frail	Age	11.15	8.51	1.31	0.19
Pre-frail—Frail		36.62	8.42	4.35	0.00
Non-frail—Frail		25.47	8.60	2.96	0.00
Frail—Pre-frail	Height (cm^2^)	−8.00	8.42	−0.95	0.34
Frail—Non-frail		−22.08	8.60	−2.57	0.01
Pre-frail—Non-frail		14.08	8.51	1.65	0.10

**Table 6:** Analysis of sociodemographic and clinical variables influencing frailty status in patients with cardiac conditions.

Clinical and Sociodemographic Variables	β Error	Standard Error	Wald	df	*p*	Exp(β) (Odds Ratio)	95% CI for Exp(β)	
							Lower limit	Upper limit
Age	0.108	0.026	16.734	1	0.000	1.114	1.058	1.173
Sex (female)	0.278	0.371	0.561	1	0.454	1.320	0.638	2.729
Sex (male)	−0.278	0.371	0.561	1	0.454	0.758	0.366	1.566
Risk of falls (YES)	−0.427	0.836	0.261	1	0.609	0.652	0.127	3.360
Downton score	0.448	0.155	8.385	1	0.004	1.565	1.156	2.120
Readmission	0.934	0.499	3.502	1	0.061	2.544	0.957	6.764
Diabetes (YES)	0.357	0.364	0.959	1	0.327	1.429	0.700	2.916
Chest pain (YES)	0.000	0.370	0.000	1	1.000	1.000	0.485	2.064
Dyspnoea (YES)	0.480	0.376	1.624	1	0.203	1.615	0.773	3.378
Barthel index	−0.103	0.028	13.503	1	0.000	0.902	0.854	0.953
Oxygen saturation (SpO_2_)	−0.140	0.073	3.713	1	0.054	0.869	0.754	1.002
Heart rate	0.001	0.011	0.003	1	0.960	1.001	0.980	1.022
Systolic blood pressure	0.006	0.009	0.385	1	0.535	1.006	0.988	1.024
Diastolic blood pressure	−0.037	0.018	4.247	1	0.039	0.963	0.930	0.998
Blood glucose	0.001	0.003	0.158	1	0.691	1.001	0.995	1.008
Length of stay	0.096	0.038	6.318	1	0.012	1.101	1.021	1.186
Waist circumference	0.031	0.015	4.352	1	0.037	1.031	1.002	1.062
BMI	0.061	0.041	2.225	1	0.136	1.063	0.981	1.152

**Table 7:** Relationship between frailty status (frail–non-frail) and the Downton scale items.

J.H. Downton Scale Items	β Error	Standard Error	Wald	df	*p*	Exp(β) (Odds Ratio)	95% CI for Exp(β)	
							Inferior	Superior
Previous falls (YES)	0.000	0.535	0.000	1	1.000	1.000	0.351	2.851
Medications (diuretics)	1.447	0.680	4.525	1	0.033	4.250	1.120	16.120
Sensory deficit (extremities)	2.084	1.178	3.130	1	0.077	8.040	0.799	80.943
Mental state (confused)	21.917	40,192.969	0.000	1	1.000	3,299,693,296.036	0.000	
Gait (normal)	−1.361	0.474	8.249	1	0.004	0.256	0.101	0.649

**Table 8:** Relationship between frailty status (frail–non-frail) and clinical diagnoses at admission.

Diagnoses	β Error	Standard Error	Wald	df	*p*	Exp(β) (Odds Ratio)	95% CI for Exp(β)	
							Lower limit	Upper limit
Arrhythmias	−1.386	0.894	2.402	1	0.121	0.250	0.043	1.443
Infectious endocarditis	−0.693	1.581	0.192	1	0.661	0.500	0.023	11.088
Heart failure	−1.041	0.801	1.689	1	0.194	0.353	0.073	1.698
Coronary heart disease	−1.732	0.748	5.368	1	0.021	0.177	0.041	0.766
Heart transplant	20.510	40,192.969	0.000	1	1.000	807,737,421.426	0.000	

## Data Availability

The data presented in this study are available upon request from the corresponding author.

## Public Involvement Statement

No public involvement in any aspect of this research.

## Guidelines and Standards Statement

This manuscript was drafted in alignment with the STROBE (Strengthening the Reporting of Observational Studies in Epidemiology) guidelines for observational research [43].

## Abbreviations

The following abbreviations are used in this manuscript:

CVDs	Cardiovascular diseases
BPSO^®^	Best Practice Spotlight Organisations
RR	Relative risk
SBP	Systolic blood pressure
DBP	Diastolic blood pressure
HR	Heart rate
SpO_2_	Oxygen saturation
BMI	Body mass index
RedCAP	Research Electronic Data Capture
ECRmp	Ethics Committee for Research with medicinal products
SD	Standard deviation
OR	Odds ratio
CI	Confidence interval
HR	Hazard ratio
